# Easy as piadcs: A low‐cost, ultra‐high‐resolution data acquisition system using a Raspberry Pi

**DOI:** 10.1002/aps3.11485

**Published:** 2022-06-08

**Authors:** Anna Knapp, Arnold J. Bloom

**Affiliations:** ^1^ Department of Plant Sciences, University of California Davis, One Shields Avenue Davis California 95616 USA

**Keywords:** data acquisition, Go, high resolution, Python, Raspberry Pi, thermocouple

## Abstract

**Premise:**

High‐precision data acquisition (DAQ) is essential for developing new methods in the plant sciences. Commercial high‐resolution DAQ systems are cost prohibitive, whereas the less expensive systems that are currently available lack the resolution and precision required for many physiological measurements.

**Methods and Results:**

We developed the software libraries, called piadcs, and hardware design for a DAQ system based on an ultra‐high‐resolution analog‐to‐digital converter and a Raspberry Pi computer. We tested the system precision with and without a thermocouple attached and found the precision with the sensor to be better than ±0.01°C and the maximum possible system resolution to be 0.4 ppm.

**Conclusions:**

The ultra‐high‐resolution DAQ system described here is inexpensive, flexible enough to be used with many different sensors, and can be built by researchers with rudimentary electronic and computer skills. This system is most applicable in the development of new measurement techniques and the improvement of existing methods.

Most studies that monitor plants and their environment, whether it be in the field or in the laboratory, require sensors that convert physical or chemical energy into an electrical signal. Some examples of sensors commonly used in plant research are thermocouples, which convert temperature gradients into an electrical potential; photodiodes, which convert light into an electrical current; and strain gauges, which have an electrical resistance that changes when deformed. Many existing methods, such as sap flow measurement (Smith and Allen, 1993), measuring chloroplast movement (Königer et al., 2021), and lysimeters (Payero and Irmak, 2008), utilize these types of sensors, and nearly all methods that use sensors require a data acquisition (DAQ) system to record measurements. Such systems usually have two basic components: (1) an analog‐to‐digital converter (ADC) that converts the electrical signal from the sensor into digital information and (2) a microcontroller or computer that records and processes the digital information from the ADC (Emilio, 2013). There are many commercially available DAQ systems, but these products are often expensive and lack flexibility; a project may need a custom DAQ system to overcome these limitations. One ideal choice for a custom system is a Raspberry Pi computer paired with a high‐resolution ADC. The low cost, flexibility, and high resolution of such a system is ideal for improving existing plant research methods or for developing new ones.

The Raspberry Pi (https://www.raspberrypi.org; Raspberry Pi Foundation, Cambridge, United Kingdom) is an inexpensive, single‐board computer that has many easily accessible and configurable input/output (I/O) interfaces, including multiple serial peripheral interfaces (SPI) and general‐purpose input/output pins (GPIO), which allow it to be used with a wide variety of ADCs and other peripheral devices. It can run many different operating systems, but the most common is the Linux‐based Raspberry Pi OS (https://www.raspberrypi.org/software/operating-systems/), which supports most programming languages. The Raspberry Pi and other similar single‐board computers have many possible applications in life science research. Its small size and low cost make it suitable for data logging in a variety of environments. The easily accessed I/O interfaces can be connected to many different types of sensors for data acquisition, including cameras for high‐throughput plant imaging (Tovar et al., 2018), microphones for bioacoustic data collection (Whytock and Christie, 2017), or gas sensors for air quality monitoring (Suriano, 2021). These same interfaces can also be used to control external components such as mechanical actuators, lighting, or temperature control.

To use sensors for data logging with a Raspberry Pi, an ADC is needed to convert the analog output of a sensor into digital information that the computer can use. Many different ADCs are available for this purpose, and it is important to choose one that is appropriate for the application. A few important specifications to consider when choosing an ADC are bit resolution, sampling rate, and number of channels. There is a necessary tradeoff between an ADC's sampling rate and effective resolution, in that ADCs with very high resolutions are limited to sampling rates in the kilohertz range or less and that as the sampling rate of a given ADC is increased the effective resolution declines (Beev, 2018). For applications where ultra‐high‐resolution is not critical, there are many ADCs on the market that have readily available open‐source software libraries and schematics for interfacing with a Raspberry Pi. For example, Adafruit (https://www.adafruit.com; Adafruit Industries, New York, New York, USA) sells ADCs with resolutions ranging from 8‐ to 16‐bits and includes open‐source software libraries and hardware design for interfacing them with a Raspberry Pi or other single‐board computer. Some sensing applications, however, such as thermocouple psychrometry and load cell measurement, involve the detection of small changes within a large measurement range. These applications may require higher than 16‐bit resolution, thus necessitating an ADC with higher resolution along with low‐noise and low‐drift electronic components.

The DAQ system described here provides high resolution at a significantly lower cost (around US$100) than commercial laboratory DAQ systems with similar specifications. It uses the ADS1262 (or ADS1263) ADC (Texas Instruments, Dallas, Texas, USA), which has 32‐bit resolution, very low noise and drift (0.16 µV peak‐to‐peak noise and 1 nV/°C offset voltage drift), as well as many built‐in features (e.g., 10‐channel multiplexer, programmable‐gain amplifier, temperature sensor for thermocouple cold junction reference, stable voltage reference, digital filters). The ADS1262 and ADS1263 are identical except that the ADS1263 also includes one additional independently controlled 24‐bit ADC. The ADS1262 was used in the system described here, but the ADS1263 can also be used and will perform the same. These features allow this ADC to be used with many different types of sensors; however, the manufacturer of this ADC does not provide a software library that allows it to be easily interfaced with a Linux computer like the Raspberry Pi. To address this, we describe here the open‐source software libraries we developed to provide this interface, called piadcs, and the electronic system design required to use this ADC with a Raspberry Pi to make ultra‐high‐resolution measurements.

## METHODS AND RESULTS

### Basic description

**Figure 1:**
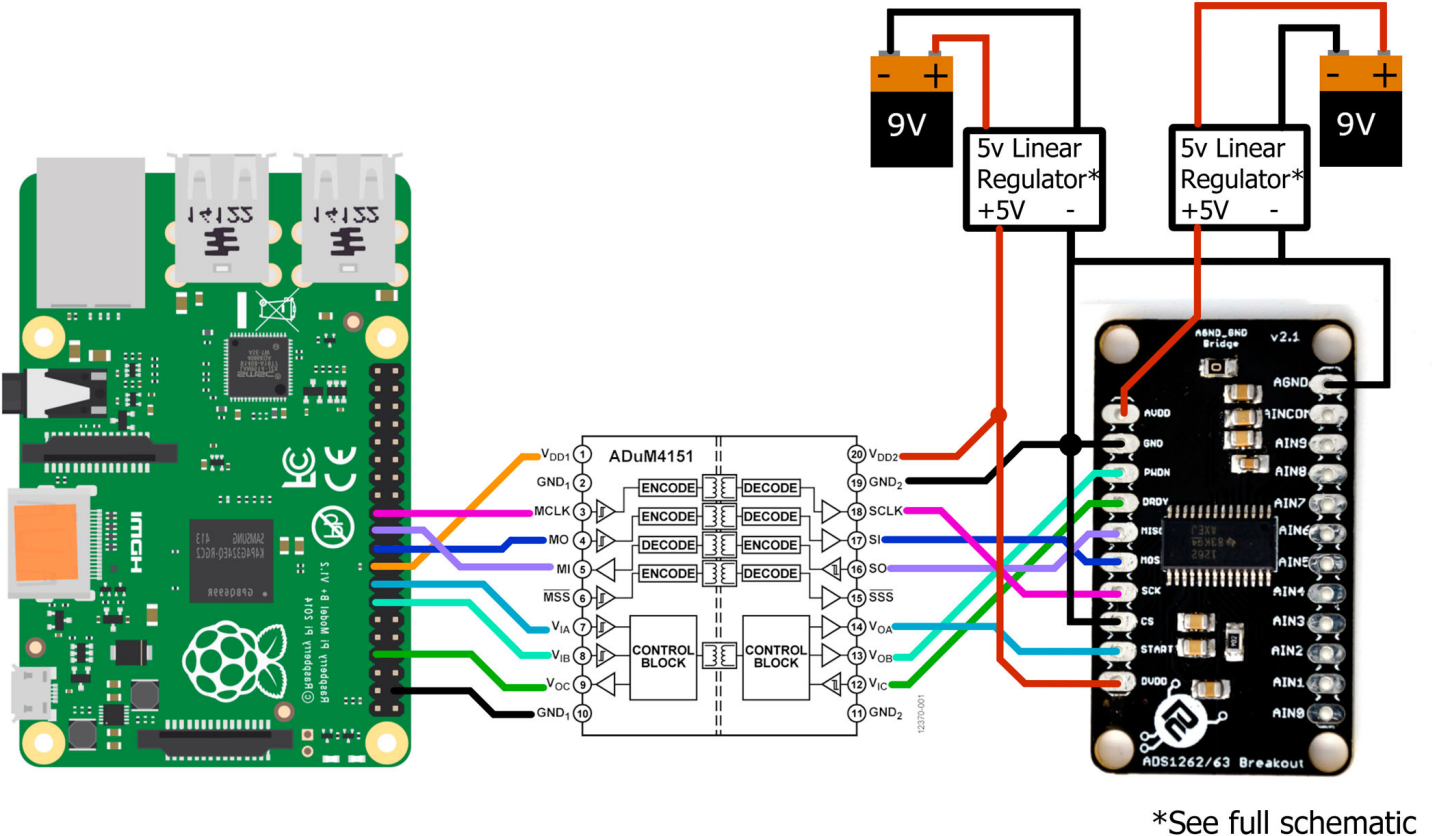
Wiring diagram for the low‐cost custom data acquisition (DAQ) system that was used to test the piadcs libraries. The ADS1262 is powered using separate power supplies for the analog and digital components. The analog power supply uses the µA723 150‐mA, 40‐V, adjustable linear voltage regulator (Texas Instruments), and the digital supply uses the LM10 operational amplifier (Texas Instruments) configured as a voltage regulator. The ADS1262 is connected through a ProtoCentral breakout board (https://protocentral.com/product/protocentral-ads1262-32-bit-precision-adc-breakout-board/) and interfaces with a Raspberry Pi 4 Model B through the ADuM4151 7‐channel SPIsolator (Analog Devices). Materials to build this DAQ system cost between US$80 and US$120 depending on which version of the Raspberry Pi 4 Model B is used. The full schematic, including the power supply circuit, is available on GitHub (https://github.com/AnnaKnapp/piadcs).

The DAQ system consists of a relatively simple hardware design based around a Raspberry Pi and the ADS1262/3 (Table 1, Figure 1), and the piadcs software libraries enable users to easily configure the ADS1262/3 and to collect, convert, and store the output data. The ADS1262/3 is a good choice for a custom DAQ system because of its extremely high (32‐bit) resolution and many features that give it flexibility (e.g., a programmable gain amplifier [PGA] with six different gain options, five different digital filters, and a range of 16 possible sampling frequencies from 2.5 to 38,000 samples/second). Functions to modify these settings are found in both piadcs libraries. The libraries also read the ADC output data and convert it into voltage values. There are functions for both reading data continuously or reading on command.

**Table 1:** Materials needed to build the piadcs data acquisition (DAQ) system.

Materials	Purpose	Cost^a^	Supplier(s)
Raspberry Pi 4 Model B	Main computer. Controls and reads data from the ADC. Stores and/or displays output.	$35–$75^b^	Many. See https://www.raspberrypi.com/products/ for a list of approved suppliers for every region
32‐bit ADS1262/3 ADC with breakout board	Converts input voltage into digital information to be read by the Raspberry Pi.	$30–$44	ProtoCentral (https://protocentral.com) or, alternatively, Olimex (https://www.olimex.com)
ADuM4151 SPIsolator	Electrically isolates the ADC from the Raspberry Pi. This is important for reducing noise.	$11	Digi‐Key Electronics (https://www.digikey.com) Mouser Electronics (https://www.mouser.com)
5 V low‐dropout (LDO) voltage regulator^c^	Provides stable and low noise 5 V output to the ADC.	$4–10^d^	Digi‐Key Electronics (https://www.digikey.com) Mouser Electronics (https://www.mouser.com)
9 V batteries	Power source for the ADC. Batteries are preferable for making low‐noise measurements because there is no 60 Hz AC signal to contend with.	$4	Many

Abbrevations: ADC, analog‐to‐digital converter; SPI, serial peripheral interface.

^a^ Costs are given in U.S. dollars (US$) at the time of publication.

^b^ Varies depending on RAM option selected. The Raspberry Pi 4 Model B comes in 2, 4, and 8 GB RAM options. All three options are appropriate for use in this DAQ system.

^c^ There are multiple parts available that could serve this purpose. Examples include MIC2954, MAX883, and µA723. Each of these voltage regulators requires the use of one or more capacitors. Please refer to the datasheet for specific information.

^d^ Varies depending on which component is used. Two voltage regulators are required.

Communication between the Raspberry Pi and the ADS1262/3 uses a combination of SPI and GPIO. The Go version of the library uses the *Periph* library (https://periph.io) to control the SPI and GPIO interfaces, and the Python version uses *Spidev* (https://pypi.org/project/spidev) and *RPi.GPIO* (https://pypi.org/project/RPi.GPIO). There are other ADCs (e.g., ADS1248, ADS1283) on the market that use highly similar programming to the ADS1262/3, and the piadcs libraries are extendable to such ADCs. The libraries also contain documentation and examples that provide a template for programming other ADCs.

### Using the piadcs libraries

There are two functionally equivalent versions of the piadcs library: one is written in Python and the other in Go. The two versions offer different advantages and disadvantages due to differences between the two languages. Python is one of the most widely used programming languages, but as an interpreted language, it runs slower and does not have support for concurrency. Go is less commonly used but still among the top 20 most‐used programming languages (TIOBE, 2021). Go is a compiled language, which provides performance advantages over Python and is simpler to read than other compiled languages (e.g., C).

Both versions of the piadcs library are available on GitHub. The Go version can be found at https://github.com/AnnaKnapp/piadcs and the Python version at https://github.com/AnnaKnapp/python_piadcs. Both are installed as packages, and detailed instructions for installation and usage can be found in the README file on GitHub. In brief, the Python library is installed from the command line using the “pip3” command and the Go library is installed using the “go get” command. The “Examples” folders found in the libraries contain code examples showing how to use the different functions in the library to change ADC settings and collect data from the ADC. Documentation for the Go library can be found at https://pkg.go.dev/github.com/AnnaKnapp/piadcs and for the Python library at https://annaknapp.github.io/python_piadcs/.

These libraries were designed to run on a Raspberry Pi model 4B or 3B+ running Raspberry Pi OS. It may also be possible to run them on other models provided they are running an up‐to‐date version of the same operating system, but we have not tested this. There are many helpful guides available on how to get started with a Raspberry Pi and the Raspberry Pi OS. For an official source, the documentation section of the Raspberry Pi website provides a detailed guide (https://www.raspberrypi.com/documentation/).

### Hardware design

The DAQ system can be built using the materials listed in Table 1 for about US$100. The wiring diagram shown in Figure 1 illustrates how the components are connected. The ADS1262/3 can be connected to the Raspberry Pi using one SPI bus and three GPIO pins. In our setup (Figure 1), they are connected via the Raspberry Pi's SPI_0 and GPIO pins 4, 22, and 27, but any of the available SPI interfaces and GPIO pins could be used. These connections must be specified in the code. Several breakout boards are available for connecting the ADS1262/3 to solderless breadboards for prototyping. A breakout board from ProtoCentral (https://protocentral.com/product/protocentral-ads1262-32-bit-precision-adc-breakout-board/; ProtoCentral Electronics, Bengaluru, India) was used in the development of these libraries, but an alternative from Olimex (https://www.olimex.com; Olimex, Plovdiv, Bulgaria) would also be suitable (Table 1). A Raspberry Pi–specific “HAT” for the ADS1263 is available from Waveshare (https://www.waveshare.com/18983.htm; Waveshare Electronics, Shenzhen, China). It is compatible with the piadcs libraries, but it is not suitable for low‐noise measurements in that one cannot electrically isolate the Raspberry Pi and the ADC because they share the same power supply; moreover, this board situates heat‐generating components near the temperature sensor of the ADC.

**Figure 2:**
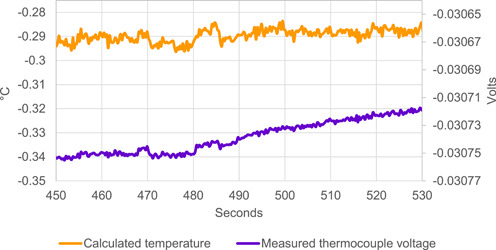
Measurement of a K‐type thermocouple in an ice bath using the custom DAQ system shown in Figure 1. The code used to take this measurement was written using the piadcs libraries and can be found in the Examples folder on the piadcs GitHub page as “typeKthermocouple.go” (https://github.com/AnnaKnapp/piadcs). The temperature was calculated from the voltage using the K‐type thermocouple polynomials provided by the National Institute of Standards and Technology (Garrity, 2000). The temperature sensor built into the ADS1262 was used for cold‐junction compensation. The measurement is slightly less than 0°C due to impurities in the water and the rough calibration value that was used to adjust for variation in the thermocouple.

High‐resolution measurements (>18‐bits) require low system noise. In our DAQ system, low noise is achieved by electrically isolating the ADS1262/3 from the Raspberry Pi. This requires separate power supplies. The Raspberry Pi is powered by a standard USB‐C wall adapter (5.1 V, 3 A), which is usually sold with the computer, whereas the ADS1262/3 is powered by 9‐V batteries connected to linear voltage regulators. Batteries are preferable to wall supplies because they do not generate any 60 Hz (or 50 Hz) AC noise. The ADS1262/3 draws less than 6.5 mA, and so a battery lasts for several weeks. The digital communication channels between the ADS1262/3 and the Raspberry Pi are also isolated using the ADuM4151 7‐channel SPIsolator (Analog Devices, Wilmington, Massachusetts, USA) (Tsemenko, 2016). All the components for this custom DAQ system can be wired onto a solderless breadboard or made into a printed circuit board. A solderless breadboard setup was used for the test measurement shown in Figure 2.

### System performance

It is possible to make very low noise measurements with this DAQ system as long as the aforementioned electrical considerations are addressed. System performance was tested with and without sensors connected. We first measured baseline system noise with no sensors connected and found that, at slower data rates (≤20 samples/second), the system noise is remarkably low (Table 2) and the system has better than 1 ppm precision. As the data rate increases, the noise increases and precision decreases somewhat but is still very good (e.g., at 7200 samples/second, the precision was 12 ppm). The ADS1262/3 has 16 different data rates available ranging from 2.5 to 38,400 samples/second, but our DAQ system only performs well at data rates up to 14,400 samples/second. This is due to a breakdown in serial communication that occurs at higher speeds and could potentially be solved in future releases.

**Table 2:** Specifications for the custom DAQ system using a Raspberry Pi 4 Model B, the ADS1262/3 analog‐to‐digital converter (ADC), and the piadcs Go library.

Specification	Typical	Maximum	Unit
Raspberry Pi 4 current draw^a^	600	1200	mA
ADS1262/3 power consumption	25	37	mW
Maximum sampling rate^b^	14,400		sps
Estimated cost of components^c^	$80–120		USD
	Data rate = 20 sps	Data rate = 7200 sps	
Peak‐to‐peak noise^d^ (PGA set to 1 V/V)	2	60	µV
System precision^e^ (PGA set to 1 V/V)	0.4	12	ppm
Peak‐to‐peak noise^f^ (PGA set to 32 V/V)	0.125	3.75	µV
System precision (PGA set to 32 V/V)	0.8	24	ppm

Abbreviations: PGA, programmable gain amplifier; sps, samples/second.

^a^ More information about Raspberry Pi power consumption can be found here https://www.raspberrypi.org/documentation/hardware/raspberrypi/power/README.md.

^b^ The ADS1262/3 ADCs are capable of higher sampling rates. Rates above 14,400 sps were not achieved in testing with the system described here due to limitations of the current release of the piadcs Go library.

^c^ Costs are given in U.S. dollars (USD) at the time of publication.

^d^ This does not include sensor noise. It was measured by shorting the analog inputs together. The digital filter was set to Sinc4 for these measurements.

^e^ This is calculated by dividing the system noise over the full measurement range.

^f^ For comparison, a Campbell Scientific CR3000 (Campbell Scientific, Logan, Utah, USA) has a maximum resolution of 0.67 µV and a noise level of about 1.3 µV peak to peak (https://s.campbellsci.com/documents/us/product-brochures/s_cr3000.pdf). A Measurement Computing MCC134 (Measurement Computing, Norton, Massachusetts, USA) has a temperature noise of 1°C (https://www.mccdaq.com/PDFs/specs/DS-MCC-134.pdf).

Although the ADC used in this system has a nominal resolution of 32‐bits, the actual system precision is lower, especially as the data rate increases. Noise remaining in the system (e.g., thermal noise), along with the inherent tradeoff between ADC speed and resolution, causes the effective resolution of an ADC to be lower than its nominal resolution (Beev, 2018). The noise floor and, therefore, the effective resolution of our system is very close to that specified in the ADS1262 datasheet for the data rates and digital filters tested (Table 2), and is a major improvement over other existing open‐source DAQ systems for the Raspberry Pi.

System performance with a connected sensor was tested by measuring the output of a K‐type thermocouple submerged in an ice bath using our DAQ system. This setup was able to measure the ice bath temperature with a noise level of less than ±0.01°C (Figure 2); this was achieved using only the analog front end provided on the ADS1262 with the PGA set to the maximum setting of 32 V/V. An external ultra‐low‐noise amplifier set to a higher gain could be used instead of the onboard PGA to further decrease noise for applications requiring very low‐level measurements (e.g., thermocouple psychrometry).

Our DAQ system has significantly better noise performance than other Raspberry Pi thermocouple DAQ systems. For example, the MCC 134 Thermocouple DAQ HAT for Raspberry Pi (Measurement Computing, Norton, Massachusetts, USA) has greater than 0.5°C measurement error with the same type of thermocouple. This is likely due partly to large thermal gradients caused by placing the DAQ board on top of the heat‐generating components of the Raspberry Pi.

## CONCLUSIONS

The greater availability of low‐cost, open‐source hardware and software makes it more feasible than ever for researchers to develop new methods for measurement and to improve on existing ones. The piadcs libraries and the hardware described here provide a simple and inexpensive way to build a Raspberry Pi–based DAQ system at a much higher resolution than was previously available using existing open‐source hardware and software. The libraries contain easy‐to‐use functions that allow for many different modes of data collection. The wiring shown in the schematic examples can be reproduced easily without extensive equipment or engineering expertise.

The system is flexible and could be used to increase the resolution of existing Raspberry Pi–based systems for monitoring plants and their environment or serve as a substantially lower‐cost alternative to expensive laboratory DAQ systems for precision laboratory measurements that necessitate high resolution, low noise, and low temperature drift. Currently, the system is best suited for laboratory applications such as growth chambers and greenhouses. It is compact and could be made portable for use in the field, but the Raspberry Pi's relatively high power consumption makes it unsuitable for long‐term data logging where power outlets are not available. Lower‐power single‐board controllers on the market such as the Raspberry Pi Pico or Arduino (https://www.arduino.cc/) could potentially be used for this purpose.

## AUTHOR CONTRIBUTIONS

A.K. and A.J.B. worked together formulating the idea for the system, A.K. wrote the software, designed the hardware, and tested it under supervision from A.J.B. A.J.B. acquired the funding for the project. A.K. wrote the original draft, and A.J.B. edited and revised it. Both authors approved the final version of the manuscript.

### OPEN RESEARCH BADGES

This article has been awarded an Open Materials badge. All materials are publicly accessible via the Open Science Framework at https://github.com/AnnaKnapp/piadcs and https://github.com/AnnaKnapp/python_piadcs. Learn more about the Open Practices badges from the Center for Open Science: https://osf.io/tvyxz/wiki.

## Data Availability

Both versions of the piadcs library, as well as detailed instructions for installation and usage, are available on GitHub (Go version: https://github.com/AnnaKnapp/piadcs; Python version: https://github.com/AnnaKnapp/python_piadcs). Documentation for the Go library can be found at https://pkg.go.dev/github.com/AnnaKnapp/piadcs and for the Python library at https://annaknapp.github.io/python_piadcs/.
